# Placebo-controlled randomised trial with liraglutide on magnetic resonance endpoints in individuals with type 2 diabetes: a pre-specified secondary study on ectopic fat accumulation

**DOI:** 10.1007/s00125-019-05021-6

**Published:** 2019-11-05

**Authors:** Maurice B. Bizino, Ingrid M. Jazet, Paul de Heer, Huub J. van Eyk, Ilona A. Dekkers, Patrick C. N. Rensen, Elisabeth H. M. Paiman, Hildebrandus J. Lamb, Johannes W. Smit

**Affiliations:** 1 Department of Radiology, Leiden University Medical Center, LUMC postzone C2S, Albinusdreef 2, 2333 ZA Leiden, the Netherlands; 2 Department of Medicine, Division of Endocrinology, Leiden University Medical Center, LUMC post zone C7Q, Albinusdreef 2, 2333 ZA Leiden, the Netherlands; 3 Einthoven Laboratory for Experimental Vascular Medicine, Leiden University Medical Center, Leiden, the Netherlands; 4 Department of Medicine, Radboud University Medical Center, Nijmegen, the Netherlands

**Keywords:** Ectopic fat, Glucagon-like peptide-1 receptor agonist, Hepatic steatosis, Liraglutide, Myocardial steatosis, Non-alcoholic fatty liver disease, Type 2 diabetes mellitus

## Abstract

**Aims/hypothesis:**

The aim of this work was to assess the effect of liraglutide on ectopic fat accumulation in individuals with type 2 diabetes mellitus.

**Methods:**

This study is a pre-specified subanalysis of the MAGNetic resonance Assessment of VICTOza efficacy in the Regression of cardiovascular dysfunction In type 2 diAbetes mellitus (MAGNA VICTORIA) study, with primary endpoints being the effects of liraglutide on left ventricular diastolic and systolic function. The MAGNA VICTORIA study was a single-centre, parallel-group trial in 50 individuals with type 2 diabetes mellitus (BMI >25 kg/m^2^) who were randomly assigned (1:1, stratified for sex and insulin use) to receive liraglutide 1.8 mg once daily or placebo for 26 weeks, added to standard care. Participants, study personnel and outcome assessors were blinded to treatment allocation. The secondary endpoints of visceral adipose tissue (VAT), abdominal subcutaneous adipose tissue (SAT) and epicardial fat were measured with MRI. Hepatic triacylglycerol content (HTGC) and myocardial triacylglycerol content (MTGC) were quantified with proton MR spectroscopy. Between-group differences (change from baseline) were tested for significance using ANCOVA. Mean differences with 95% CIs were reported.

**Results:**

The trial was completed in 2016. Twenty-four participants were randomised to receive liraglutide and 26 to receive placebo. One patient in the liraglutide group withdrew consent before having received the study drug and was not included in the intention-to-treat analysis. Liraglutide (*n* = 23) vs placebo (*n* = 26) significantly reduced body weight (liraglutide 98.4 ± 13.8 kg to 94.3 ± 14.9 kg; placebo 94.5 ± 13.1 kg to 93.9 ± 13.2 kg; estimated treatment effect −4.5 [95% CI −6.4, −2.6] kg). HbA_1c_ declined in both groups without a significant treatment effect of liraglutide vs placebo (liraglutide 66.7 ± 11.5 mmol/mol to 55.0 ± 13.2 mmol/mol [8.4 ± 1.1% to 7.3 ± 1.2%]; placebo 64.7 ± 10.2 mmol/mol to 56.9 ± 6.9 mmol/mol [8.2 ± 1.0% to 7.5 ± 0.7%]; estimated treatment effect −2.9 [95% CI −8.1, 2.3] mmol/mol or −0.3 [95% CI −0.8, 0.2]%). VAT did not change significantly between groups (liraglutide 207 ± 87 cm^2^ to 203 ± 88 cm^2^; placebo 204 ± 63 cm^2^ to 200 ± 55 cm^2^; estimated treatment effect −7 [95% CI −24, 10] cm^2^), while SAT was reduced by a significantly greater extent with liraglutide than with placebo (liraglutide 361 ± 142 cm^2^ to 339 ± 131 cm^2^; placebo 329 ± 107 cm^2^ to 333 ± 125 cm^2^; estimated treatment effect −29 [95% CI −51, −8] cm^2^). Epicardial fat did not change significantly between groups (liraglutide 8.9 ± 4.3 cm^2^ to 9.1 ± 4.7 cm^2^; placebo 9.6 ± 4.1 cm^2^ to 9.6 ± 4.6 cm^2^; estimated treatment effect 0.2 [95% CI −1.5, 1.8] cm^2^). Change in HTGC was not different between groups (liraglutide 18.1 ± 11.2% to 12.0 ± 7.7%; placebo 18.4 ± 9.4% to 14.7 ± 10.0%; estimated treatment effect −2.1 [95% CI −5.3, 1.0]%). MTGC was not different after treatment with liraglutide (1.5 ± 0.6% to 1.2 ± 0.6%) vs placebo (1.3 ± 0.5% to 1.2 ± 0.6%), with an estimated treatment effect of −0.1 (95% CI −0.4, 0.2)%. There were no adjudicated serious adverse events.

**Conclusions/interpretation:**

Compared with placebo, liraglutide-treated participants lost significantly more body weight. Liraglutide primarily reduced subcutaneous fat but not visceral, hepatic, myocardial or epicardial fat. Future larger studies are needed to confirm the results of this secondary endpoint study.

**Trial registration:**

ClinicalTrials.gov NCT01761318.

**Funding:**

This study was funded by Novo Nordisk A/S (Bagsvaerd, Denmark).

**Electronic supplementary material:**

The online version of this article (10.1007/s00125-019-05021-6) contains peer-reviewed but unedited supplementary material, which is available to authorised users.

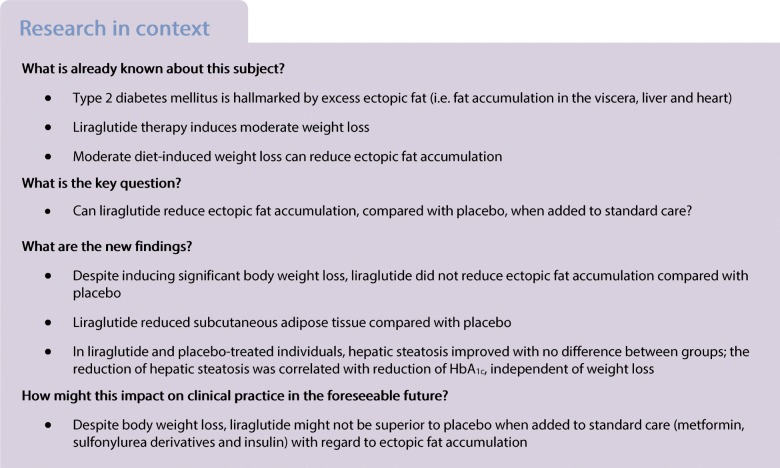



## Introduction

Insulin resistance and type 2 diabetes mellitus are hallmarked by excess fat storage in visceral adipose tissue (VAT), liver, skeletal muscle, myocardial tissue and epicardial fat [1]. VAT is tightly linked to insulin resistance and cardiovascular disease, independent of general obesity [1]. Consequently, diet-induced reduction of VAT has greater impact on markers of insulin sensitivity and cardiometabolic risk than reduction of subcutaneous adipose tissue (SAT) [2]. Hepatic steatosis is an independent predictor of cardiovascular disease, possibly by contributing to hepatic insulin resistance resulting in atherogenic dyslipidaemia [1]. Excess hepatic fat accumulation can damage the liver itself when uncomplicated steatosis progresses to non-alcoholic steatohepatitis (NASH). In parallel, myocardial steatosis and excess epicardial fat are postulated to negatively affect myocardial function and coronary vasculature, respectively [3]. Therefore, therapeutic interventions aimed at reducing excess ectopic fat storage might have a major impact on the cardiovascular prognosis of individuals with type 2 diabetes mellitus.

Liraglutide is a glucagon-like peptide-1 (GLP-1) receptor agonist (GLP-1RA) commonly used to achieve glycaemic control in individuals with type 2 diabetes. Liraglutide’s actions include stimulation of glucose-dependent insulin release from beta cells, and promotion of satiety resulting in reduced energy intake and modest weight loss. Since modest weight loss, at least by energy restriction, is associated with a reduction in ectopic fat accumulation [4], liraglutide might also have this effect. In addition, it has been shown in animal models that endogenous GLP-1 or GLP-1RAs exert pleiotropic actions in organs such as the liver and heart, and in animal studies it has been found that GLP-1 receptor (GLP-1R) agonism can decrease hepatic [5–7] and myocardial [8] steatosis, independent of weight loss. In humans, studies with GLP-1RAs that assessed VAT and hepatic steatosis in individuals with type 2 diabetes have shown conflicting results, possibly related to use of different GLP-1RAs, treatment differences in control arms and varying baseline participant characteristics [9–15]. Furthermore, it has been shown that response to intervention may vary between different ectopic fat depots [16]. Therefore, an integrated assessment on a multi-organ level is required to fully establish the potency of liraglutide in combatting ectopic fat and in long-term patient outcome. Probably due to technical challenges, myocardial steatosis and epicardial fat have been incorporated as read-outs in only few studies [17, 18].

Recent technical developments have enabled non-invasive direct quantification of ectopic fat depots in humans with high accuracy using MRI and proton magnetic resonance spectroscopy (^1^H-MRS) [19]. Using these techniques, we assessed ectopic fat as a pre-specified secondary study of the previously published MAGNetic resonance Assessment of VICTO2a efficacy in the Regression of cardiovascular dysfunction In type 2 diAbetes mellitus (MAGNA VICTORIA) study [20]. The primary purpose of that randomised placebo-controlled study was to evaluate the effect of liraglutide on left ventricular diastolic and systolic function in 50 individuals with type 2 diabetes. In the liraglutide group, early diastolic filling, stroke volume and ejection fraction reduced, compared with the placebo group. In keeping with the relationship between ectopic fat and cardiac function described above, the aim of the present study was to evaluate whether liraglutide reduces visceral fat, hepatic steatosis and myocardial steatosis.

## Methods

### Study design and participants

This study was part of the MAGNA VICTORIA study, which was an investigator-initiated randomised, double-blind, assessor-blinded, placebo-controlled, single-centre clinical trial with 26 weeks follow-up [20]. Electronic supplementary material (ESM) Table 1 provides an overview of the ClinicalTrials.gov registered trial endpoints that have already been published, those reported in the present manuscript, and endpoints that will be published in future manuscripts. In short, the study aimed to include 50 participants (men and women) aged 18–69 years with BMI 25 kg/m^2^ or above and HbA_1c_ level of 53–86 mmol/mol (7.0–10.0%) despite use of metformin, and/or sulfonylurea derivative (SUD) and/or insulin. Main exclusion criteria were as follows: use of other glucose-lowering therapy; renal, hepatic or cardiovascular disease; gastric bypass surgery; chronic pancreatitis or previous acute pancreatitis; pregnancy or lactation; and MRI contra-indications. The trial was approved by the local ethics committee and performed in accordance with the principles of the revised Declaration of Helsinki. Written informed consent was obtained from all participants before study. The trial was conducted at the Leiden University Medical Center (LUMC), Leiden, the Netherlands and was registered at ClinicalTrials.gov (registration no. NCT01761318).

### Randomisation and treatment

Included participants were randomised (1:1, stratification for sex and insulin use) to receive liraglutide (Victoza; Novo Nordisk, Bagsvaerd, Denmark) or placebo (provided by Novo Nordisk). The study drug was up-titrated to 1.8 mg once daily from week 3 onwards. The dose was reduced if necessitated by adverse events. During the study, blood-glucose-lowering drugs were titrated according to clinical practice guidelines by means of dose adjustment of SUD and/or insulin.

### Blood examinations

At study entry and at 26 weeks, blood examinations were performed after participants had fasted for at least 6 h. HbA_1c_ was measured using boronate affinity high-performance liquid chromatography (Primus Ultra; Siemens Healthcare Diagnostics, Breda, the Netherlands) throughout the first part of the study, and changed to measurement using ion-exchange high-performance liquid chromatography (Tosoh G8; Sysmex Nederland, Etten-Leur, the Netherlands) for subsequent measurements. HbA_1c_ values assessed by the boronate affinity method were corrected on the basis of the correlation coefficient derived from a validation experiment that used data of 196 samples measured on both analysers. All other blood samples were processed and analysed as described previously [20]. Adiponectin, aspartate aminotransferase (AST), alanine aminotransferase (ALT), alkaline phosphatase and γ-glutamyl transferase (GGT) concentrations were measured with a Modular P800 analyser (Roche Diagnostics, Mannheim, Germany). Serum NEFA were measured using the NEFA C kit (Wako Diagnostics, INstruchemie, Delfzijl, the Netherlands).

### MRI protocol

All participants underwent an MRI and ^1^H-MRS protocol using a clinical 3 Tesla Ingenia whole-body MR system (Philips Medical Systems, Best, the Netherlands) at baseline and follow-up. Participants were scanned in the supine position after they had fasted for at least 6 h. The body coil was used for transmission, and reception was achieved with a 16-element anterior array and 12-element posterior array. A 3D breath-hold dual-echo mDIXON sequence of the abdomen was performed (repetition time 3.5 ms; first echo time 1.19 ms; second echo time 2.3 ms; flip angle 10°; spatial resolution 16 × 17 mm; slice thickness 4 mm; slice gap 2 mm) with transverse slice orientation. Using MASS software (LUMC, Leiden, the Netherlands), post-processing involved generation of three 10 mm transverse slices with 2 mm slice gap at the level of the fourth and fifth lumbar vertebral bodies. Semi-automated segmentation of VAT and abdominal SAT was depicted by threshold-based inclusion of fat, with manual correction. VAT and SAT were calculated as mean area of fat in three slices. ^1^H-MRS of the liver was performed using a 20 × 20 × 20 mm voxel of interest, which was localised using a Point Resolved Spectroscopy Sequence (echo time 35 ms; repetition time 9 s for unsuppressed spectra and 3.5 s for water-suppressed spectra). The voxel was placed preferably in liver segment V, VI, VII or VIII. The position of the voxel at baseline was used to guide placement of the voxel at follow-up MRI in the same liver segment. Four signal averages were acquired without, and 32 with, water suppression using the Multiply Optimized Insensitive Suppression Train sequence. Spectra were acquired during free-breathing at end-expiration with pencil beam navigator-based respiratory triggering [21]. ^1^H-MRS of the heart was assessed as described previously [21]. The 15 × 25 × 40 mm voxel of interest was placed in the interventricular myocardial septum. Six signal averages were acquired without, and 48 with, water suppression. Otherwise, acquisition was the same as described above. Post-processing of proton spectra was performed with an in-house developed program as previously described [21], before fitting the spectra in the time-domain using the Java-based MR User Interface (version 5.0; Katholieke Universiteit Leuven, Leuven, Belgium). An ECG-gated breath-hold high-resolution water-suppressed Black-Blood Turbo Spin Echo Sequence (repetition time 1000 ms; echo time 11 ms; flip angle 90°; voxel 1.09 × 1.12 mm) in four-chamber view at end-diastole was used to image epicardial and paracardial fat. Epicardial fat was defined as the inner layer directly covering the myocardial outer surface and paracardial fat as the outer layer of fat surrounding the heart. The atrioventricular plane was set to determine the basal border. Pericardial fat was defined as the sum of epicardial and paracardial fat. All images and proton spectra were assessor blinded.

### Study endpoints

We previously reported the primary endpoints of the MAGNA VICTORIA study that involved left ventricular diastolic and systolic function [20]. The study endpoints VAT, SAT, hepatic triacylglycerol content (HTGC), myocardial triacylglycerol content (MTGC) and epicardial fat reported in the current manuscript were secondary endpoints of the MAGNA VICTORIA study. Other pre-specified endpoints were body weight, BMI, waist:hip ratio, HbA_1c_, serum triacylglycerols, NEFA, total cholesterol, HDL-cholesterol, LDL-cholesterol, adiponectin and liver enzymes. Endpoints that were not predefined were paracardial and pericardial fat.

### Statistics

Data are shown as mean ± SD when normally distributed, or as median (interquartile range) when not normally distributed. For all presented study endpoints, we performed an ANCOVA of between-group differences of change from baseline, with randomisation arm as fixed effect and baseline measurement of dependent variable as covariate. In addition, a sensitivity analysis was performed that also included sex and insulin use as covariates in the ANCOVA model, since randomisation was stratified by sex and insulin use. Mean changes from baseline ± SD are reported for within group changes, and estimated treatment effect with 95% CIs are displayed for between-group differences. In addition to these pre-specified analyses, an assessment of associations between observed change in HTGC and change in HbA_1c_ was performed. First, correlation between change in HbA_1c_ and HTGC was estimated using Pearson’s correlation. Subsequently, a stepwise multiple linear regression analysis was performed to assess the following: (1) unadjusted association of the independent variables change in HbA_1c_, sex, age, treatment group allocation and weight loss with the dependent variable change in HTGC; (2) adjusted association for the independent variables mentioned above. All statistical analyses were performed as described previously [20]. We considered a *p* value of <0.05 statistically significant.

## Results

Participants were enrolled between December 2013 and September 2015, with the final participant visit taking place in March 2016. The trial flow chart was published previously [20]. One participant in the liraglutide group withdrew consent before having received the study drug and was not included in intention-to-treat analysis, and one participant was withdrawn from the study because of frequent hypoglycaemic events (on further examination, a diagnosis of type 1 diabetes was made). In the placebo group, one participant was lost to follow-up. Intention-to-treat analysis was performed in 23 participants in the liraglutide group and 26 in placebo group. Baseline characteristics are shown in Table 1. Sex, insulin use, age, lipid levels, smoking history and glycaemic control were comparable between groups. Liraglutide recipients had slightly higher BMI. During the study, SUD and insulin doses were titrated on ambulant glucose levels and HbA_1c_ values. This resulted in decreased total use of SUDs and insulin in liraglutide-treated participants and an increase in placebo-treated participants. An overview of concomitant drug use was described previously [20].

**Table 1:** Baseline characteristics of trial population

Characteristic	Liraglutide (*n* = 23)	Placebo (*n* = 26)
Demographics		
Age, years	60 ± 6	59 ± 7
Men	14 (61)	15 (58)
Diabetes duration, years	11 ± 6	11 ± 7
Diabetic retinopathy, *n*	4 (17)	2 (8)
Diabetic nephropathy, *n*	2 (9)	11 (42)
Diabetic neuropathy, *n*	10 (44)	7 (27)
Diabetic macrovascular complications^a^, *n*	2 (9)	0 (0)
Clinical variables		
Weight, kg	98 ± 14	94 ± 13
BMI, kg/m^2^	32.6 ± 4.4	31.6 ± 3.4
Waist, cm	111 ± 10	109 ± 9
Hip, cm	108 ± 8	106 ± 7
Waist:hip ratio	1.03 ± 0.06	1.03 ± 0.08
Systolic BP, mmHg	141 ± 14	141 ± 15
Diastolic BP, mmHg	86 ± 6	87 ± 11
Triacylglycerols, mmol/l	2.2 ± 1.5	2.1 ± 1.1
NEFA, mmol/l	0.7 ± 0.2	0.8 ± 0.5
Total cholesterol, mmol/l	4.8 ± 1.0	4.8 ± 1.0
HDL-cholesterol, mmol/l	1.2 ± 0.2	1.3 ± 0.4
LDL-cholesterol, mmol/l	2.6 ± 0.9	2.5 ± 0.9
HbA_1c_, mmol/mol	67 ± 12	65 ± 10
HbA_1c_, %	8.4 ± 1.1	8.2 ± 1.0
Adiponectin, mg/l	5.5 ± 2.4	6.5 ± 3.5
AST, U/l	31 ± 11	35 ± 21
ALT, U/l	15 ± 7	13 ± 5
Alkaline phosphatase, U/l	73 ± 22	72 ± 19
GGT, U/l	38 ± 24	34 ± 19
Smoking history		
Never smoked, *n*	10 (44)	8 (31)
Current smoker, *n*	4 (17)	5 (19)
Ex-smoker, *n*	9 (39)	13 (50)
Concomitant drug use		
Metformin dose, g/day	2.1 ± 0.7	2.0 ± 0.5
Sulfonylurea, *n*	6 (26)	8 (31)
Insulin, *n*	15 (65)	17 (65)
Anti-lipidaemic drug, *n*	21 (91)	19 (73)
Anti-hypertensive drug, *n*	18 (78)	20 (77)

Data are presented as *n* (%) or mean ± SD

^a^Macrovascular complications were cerebrovascular or peripheral artery disease and not cardiovascular

### Anthropometric measurements and laboratory values

Changes in anthropometric and laboratory measures are displayed in Table 2. Liraglutide significantly decreased body weight compared with placebo (liraglutide 98.4 ± 13.8 kg to 94.3 ± 14.9 kg; placebo 94.5 ± 13.1 kg to 93.9 ± 13.2 kg). In addition, both waist and hip circumference reduced in liraglutide- vs placebo-treated participants, without a difference in waist:hip ratio. There was no difference between groups for any of the laboratory measures (see Table 2). HbA_1c_ differences from baseline were not different between groups. In liraglutide-treated participants, HbA_1c_ decreased from 66.7 ± 11.5 mmol/mol to 55.0 ± 13.2 mmol/mol (8.4 ± 1.1% to 7.3 ± 1.2%), and in placebo-treated participants HbA_1c_ decreased from 64.7 ± 10.2 mmol/mol to 56.9 ± 6.9 mmol/mol (8.2 ± 1.0% to 7.5 ± 0.7%).

**Table 2:** Within-group and between-group changes from baseline of anthropometric and laboratory measurements

Variable	Change from baseline to 26 weeks		Mean (95% CI) change from baseline (liraglutide vs placebo)	*p* value
	Liraglutide (*n* = 23)	Placebo (*n* = 26)		
Anthropometric measures				
Weight, kg	−4.3 ± 3.8	0.1 ± 2.5	−4.5 (−6.4, −2.6)	<0.001
BMI, kg/m^2^	−1.5 ± 1.3	0.1 ± 0.8	−1.5 (−2.2, −0.9)	<0.001
Waist, cm	−1 ± 4	2 ± 4	−3 (−5, −1)	0.004
Hip, cm	−2 ± 5	1 ± 2	−3 (−5, −1)	0.002
Waist:hip ratio	0.01 ± 0.04	0.01 ± 0.04	0.00 (−0.02, 0.03)	0.69
Laboratory measures				
Triacylglycerols, mmol/l	−0.5 ± 1.1	−0.61.0	0.2 (−0.1, 0.6)	0.18
NEFA, mmol/l	−0.1 ± 0.2	−0.1 ± 0.5	−0.1 (−0.2, 0.1)	0.39
Total cholesterol, mmol/l	−0.7 ± 0.9	−0.5 ± 0.6	−0.22 (−0.59, 0.15)	0.23
HDL-c, mmol/l	−0.0 ± 0.1	0.1 ± 0.3	−0.1 (−0.2, 0.1)	0.22
LDL-c, mmol/l	−0.4 ± 0.5	−0.2 ± 0.5	−0.17 (−0.44, 0.10)	0.22
HbA_1c_, mmol/mol	−11.6 ± 11.1	−7.7 ± 9.4	−2.9 (−8.1, 2.3)	0.27
HbA_1c_, %	−1.1 ± 1.0	−0.7 ± 0.9	−0.3 (−0.8, 0.2)	0.27
Adiponectin, mg/l	−0.5 ± 1.8	−0.8 ± 2.0	0.2 (−0.9, 1.4)	0.67
AST, U/l	−6 ± 11	−1 ± 22	2 (−3, 6)	0.46
ALT, U/l	16 ± 12	14 ± 10	1 (−5, 7)	0.78
AP, U/l	5 ± 11	6 ± 8	−1 (−7, 5)	0.76
GGT, U/l	1 ± 18	−3 ± 11	3 (−6, 12)	0.47

Data are presented as mean ± SD

AP, alkaline phosphatase; HDL-c, HDL-cholesterol; LDL-c, LDL-cholesterol

### Ectopic fat

The results of the MRI and MRS analysis of ectopic fat are summarised in Table 3. ESM Table 2 shows the sensitivity analysis with randomisation stratifiers sex and insulin use as additional covariates. The result of this analysis was similar to that of the primary analysis shown in Table 3. Liraglutide did not reduce VAT compared with placebo. In contrast, liraglutide significantly reduced SAT compared with placebo. The between-group changes in the VAT:SAT ratio were not different (estimated mean treatment effect 0.03 [95% CI −0.09, 0.04], *p* = 0.43). ^1^H-MRS of the liver was technically not successful on two occasions (one baseline measurement; one follow-up measurement in a different participant), and one participant in the placebo group displayed a biologically implausible rise in HTGC from 1.7% at baseline to 39.5% at follow-up without changes in serum liver enzymes. This measurement was therefore excluded from analysis (sensitivity analysis revealed no differences after exclusion of this measurement, data not shown). ^1^H-MRS of the heart was successful on all but four occasions: one at baseline due to low signal-to-noise ratio and three at follow-up due to low signal-to-noise ratio or incorrect peak frequency. With regard to MTGC, there was also no significant treatment effect of liraglutide vs placebo. With regard to epicardial, paracardial and pericardial fat, between-group differences were not significant. None of the ectopic fat variables showed significant correlation with indices of left ventricular function that had been published previously [20] (data not shown).

**Table 3:** Ectopic fat accumulation

Variable	Liraglutide (*n* = 23)			Placebo (*n* = 26)			Mean (95% CI) change from baseline (liraglutide vs placebo)	*p* value
	Baseline	26 weeks	Change from baseline	Baseline	26 weeks	Change from baseline		
VAT, cm^2^	207 ± 87	203 ± 88	−8 ± 33	204 ± 63	200 ± 55	0 ± 27	−7 (−24, 10)	0.41
SAT, cm^2^	361 ± 142	339 ± 131	−28 ± 40	329 ± 107	333 ± 125	3 ± 30	−29 (−51, −8)	0.007
HTGC, %	18.1 ± 11.2	12.0 ± 7.7	−6.3 ± 7.1	18.4 ± 9.4	14.7 ± 10.0	−4.0 ± 4.6	−2.1 (−5.3, 1.0)	0.17
MTGC, %	1.5 ± 0.6	1.2 ± 0.6	−0.3 ± 0.5	1.3 ± 0.5	1.2 ± 0.6	−0.0 ± 0.5	−0.1 (−0.4, 0.2)	0.39
Epicardial fat, cm^2^	8.9 ± 4.3	9.1 ± 4.7	0.3 ± 3.0	9.6 ± 4.1	9.6 ± 4.6	0.0 ± 2.2	0.2 (−1.5, 1.8)	0.85
Paracardial fat, cm^2^	25.7 ± 10.9	24.7 ± 10.9	−1.1 ± 6.0	20.6 ± 10.0	22.0 ± 10.3	1.4 ± 5.7	−1.8 (−5.2, 1.6)	0.28
Pericardial fat, cm^2^	34.6 ± 13.4	33.8 ± 13.9	−0.8 ± 7.4	30.2 ± 12.3	31.7 ± 12.4	1.5 ± 5.7	−1.8 (−5.9, 2.3)	0.39

Data are presented as mean ± SD

### Association between HbA_1c_ and hepatic steatosis

**Fig. 1:**
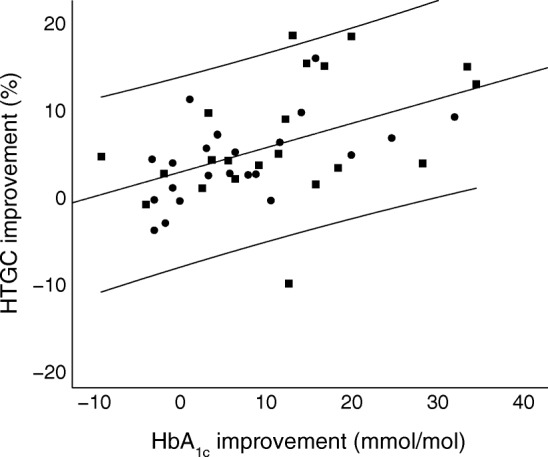
Scatter plot of HbA_1c_ vs HTGC difference (baseline – follow-up at 26 weeks) for all participants. Circles, placebo; squares, liraglutide. The unstandardised regression line (*y* = 2.57 + 0.28*x*) with 95% CI is shown

Reduction of HbA_1c_ correlated well with reduction of HTGC in the whole cohort (*r* = 0.49, *p* = 0.001). Figure 1 shows a scatterplot of the change in HbA_1c_ and change in HTGC between baseline and follow-up. The regression line had an unadjusted slope of 0.28 (95% CI 0.12, 0.44, *p* = 0.001). Stepwise multiple linear regression analysis revealed that, of the independent variables change in HbA_1c_, sex, age, treatment group allocation and weight loss, only change in HbA_1c_ significantly correlated with change in HTGC (ESM Table 3). After adjustment for sex, age, treatment group allocation and weight loss, the adjusted estimate of association for change in HbA_1c_ was 0.50 (*p* = 0.001).

## Discussion

This study shows that, compared with placebo, liraglutide reduced body weight and subcutaneous fat but not visceral fat, hepatic steatosis, myocardial steatosis, epicardial fat, paracardial fat or pericardial fat. Despite a significant 4 kg weight loss in liraglutide-treated participants over 6 months, there was no reduction of ectopic fat accumulation.

In addition to blood-glucose lowering, liraglutide decreases energy intake and lowers body weight. In view of the preferential loss of VAT by modest weight loss induced by diet [4], one would expect that liraglutide-associated weight loss would also diminish VAT. In this study, however, both waist:hip ratio and MRI assessment of abdominal fat are consistent with a preferential loss of SAT. While this result is in line with the findings of Suzuki et al., who used a dose of 0.9 mg liraglutide daily in a single-arm intervention study [14], others have shown reduction of predominantly VAT by GLP-1RA treatment [9, 11–13, 22] or no effect on SAT or VAT [10]. These studies were performed in type 2 diabetes patients with varying ethnicity, BMI and concomitant treatment regimes, making it difficult to compare results.

It is interesting to speculate on the mechanism by which liraglutide could preferentially diminish SAT. First, it can be hypothesised that liraglutide directly affects the lipogenesis:lipolysis ratio in adipose tissue by binding to GLP-1Rs in adipocytes. However, studies using validated specific methods have not been able to detect the canonical GLP-1R in adipocytes [23], so other mechanisms are more likely. Probably, the abundance of GLP-1Rs in white adipose tissue arises from expression in non-adipocyte cells, such as vascular endothelial cells, peripheral neurons and/or macrophages, which have been shown to express GLP1-Rs [23, 24]. In mice, treatment with GLP-1 affects white adipose tissue macrophage phenotype, inflammatory cytokine profile, lipogenic gene expression and fat oxidation in conjunction with increased insulin sensitivity [24]. Although these mouse studies provide a theoretical basis for a direct effect of GLP-1RAs on white adipose tissue, it is not known whether these mechanisms play a role in body fat distribution in humans. Another potential mechanism by which liraglutide could influence body fat distribution is the central nervous system. The autonomic nervous system has been shown to provide distinct innervation of VAT and SAT [25], each having its own sympathetic and parasympathetic innervations with catabolic and anabolic effects, respectively [26]. These findings, in combination with the knowledge that isolated central nervous system administration of GLP-1RAs can reduce body fat in mice with diet-induced obesity [27], provide a theoretical basis for the hypothesis that liraglutide can alter body fat distribution via distinct action on sympathetic and parasympathetic stimulation with consequent lipolytic and lipogenic action, respectively.

Targeting hepatic steatosis is an important part of type 2 diabetes mellitus management. When considering the lack of beneficial effect of liraglutide vs placebo in our study participants, one must also consider that, in addition to placebo, concomitant SUD and insulin were titrated to reach the HbA_1c_ goal <53 mmol/mol (7%). Therefore, our study is best compared with studies using active comparators against GLP-1RA treatment. In their open-label trial, Tang et al. treated individuals with type 2 diabetes with liraglutide vs insulin, resulting in comparable improvement in glycaemic control among treatment arms, and no between-group difference in liver fat fraction [15]. Bi et al. reported corresponding results in their open-label randomised trial with exenatide vs insulin [9]. In contrast to our study, several studies have shown that treatment with a GLP-1RA reduced HTGC when compared with treatment with insulin [17, 28]. Dutour et al. performed a randomised open-label study in which individuals with type 2 diabetes were treated with exenatide vs insulin and were assessed for HTGC after a standard meal and showed a 23.8 ± 9.5% relative reduction in HTGC for the exenatide group compared with a 12.5 ± 9.6% increase in the insulin group (*p* = 0.007) [17]. Yan et al. also found HTGC to be significantly decreased in liraglutide-treated but not insulin-treated individuals [28]. Although body weight and HbA_1c_ reductions in these studies were comparable with those reported in our study, an important difference was that individuals already using insulin were excluded in these studies. Whether that explains the discrepancy with our study is unclear because randomised studies comparing the effect of add-on GLP-1RA on liver fat in insulin-treated individuals are currently lacking. In a single-arm study by Petit et al., 68 individuals with type 2 diabetes (21% using insulin at baseline) were treated with liraglutide 1.2 mg once daily, resulting in a 31% RR reduction of hepatic steatosis [29]. This cohort was compared against another study cohort with comparable baseline characteristics (*n* = 16) who underwent intensification of the glucose-lowering regimen with insulin and who showed a non-significant decline of liver fat fraction. Apart from the non-randomised design, the results of this study could have been influenced by the fact that patients also received instructions on healthy diet and exercise. The only study evaluating the effect of liraglutide on histological resolution of NASH was the Liraglutide Safety and Efficacy in Patients with Non-alcoholic steatohepatitis (LEAN) trial [30]. This placebo-controlled randomised trial in 52 individuals with biopsy-proven NASH showed that liraglutide led to histological resolution of NASH. In this trial, however, only 33% of participants had type 2 diabetes and there was no active comparator, resulting in a significant improvement of HbA_1c_ in the liraglutide vs control group. In keeping with the hypothesis that reduction in HbA_1c_ is associated with reduction in HTGC, we and others [9, 15, 31] have found a significant correlation between HbA_1c_ reduction and HTGC reduction. One theory could be that improved glycaemic control directly reduced HTGC (e.g. by decreased de novo lipogenesis via carbohydrate response element binding protein [32]). However, in light of conflicting results in clinical studies, more research is needed to provide insight on this topic.

Myocardial steatosis is characterised by an increased triacylglycerol content in cardiomyocytes, as assessed by ^1^H-MRS of the heart. The abundance of intracellular triacylglycerols is associated with increased deposition of toxic lipids that interfere with cardiac energy metabolism and cell survival [19]. In individuals with type 2 diabetes, myocardial steatosis is a predictor of concentric left ventricular remodelling, impaired systolic strain and diastolic dysfunction [3, 33]. Reversal of myocardial steatosis with very-low-energy diet is associated with improved left ventricular diastolic function [34]. Based on these observations, and preclinical studies showing improved cardiac lipotoxicity in GLP-1RA-treated mice [8, 35] and in vitro protection from ceramide-induced cardiomyocyte apoptosis by GLP-1RA [36], liraglutide might have the potential to reverse myocardial steatosis in individuals with type 2 diabetes. Conversely, our data do not support this hypothesis. This is also in conjunction with the fact that liraglutide did not ameliorate left ventricular myocardial relaxation (i.e. diastolic function) in this particular study population, and that left ventricular systolic function slightly decreased, presumably in relation to decreased left ventricular preload [20]. These results are in keeping with another study performed in this particular research area [17].

Excess epicardial fat accumulation is hypothesised to exert local effects by leading to secretion of inflammatory and metabolic peptides by tissues that might contribute to coronary artery disease. Epicardial adiposity has been linked to visceral obesity [37] and coronary events [38] and is a reversible phenomenon upon weight loss [39]. However, there was no reduction in epicardial fat volume in liraglutide-treated participants in this study. As suggested by their shared origin, it might be that epicardial and visceral fat have responded likewise to liraglutide treatment [40]. Iacobellis et al. and Dutour et al. did find a significant reduction in epicardial fat [17, 18] in their open-label studies. A possible explanation for this discrepancy could be the larger weight loss in these studies, which may have been based on their non-blinded study design.

Liraglutide has been shown to have a safe cardiovascular endpoint profile, with less major cardiovascular events compared with placebo added to standard care [41]. There are many hypotheses on the mechanisms by which GLP-1RAs might reduce cardiovascular risk, including lowering of blood pressure, improved vascular endothelial function, improved lipid metabolism, reduced inflammatory profile, direct beneficial effect on the heart, and weight loss [23]. Our finding that liraglutide does not reduce ectopic fat supports the hypothesis that weight loss is not the main driver of the cardiovascular benefit of GLP-1RA therapy.

The primary limitation of this study was that the presented outcome measures were not the primary outcomes of the MAGNA VICTORIA study. This could imply that the study was underpowered with regard to evaluation of the treatment effect on ectopic fat. In that regard, the 95% CIs of between-group changes from baseline are crucial to the interpretation of this study [42]. In line with the fact that 5% mean weight loss difference between liraglutide and placebo-treated participants closely approximates the average weight loss use of liraglutide 1.8 mg in larger studies [43], it is likely that the ectopic fat changes are also representative. Obviously, given the heterogeneity of our study population, we cannot exclude the possibility that certain subgroups of individuals (e.g. those with severe hepatic steatosis) might benefit from liraglutide therapy with respect to lowering hepatic steatosis.

In conclusion, this pre-specified secondary study showed that liraglutide did not reduce ectopic fat accumulation in individuals with type 2 diabetes, compared with placebo treatment added to standard care. Tight glycaemic control in both treatment groups was associated with reduced hepatic steatosis, with no added effect of liraglutide. From a clinical perspective, weight loss caused by liraglutide therapy might not be crucial for its beneficial cardiovascular actions, wherein other mechanisms such as inflammation and lipid metabolism are probably involved [23]. Future studies are desirable to explore whether ectopic fat accumulation can be reduced with GLP-1RAs in certain subgroups, such as those with BMI >35 kg/m^2^ and/or more severe hepatic steatosis.

## Electronic supplementary material

ESM (PDF 319 kb)

## Data Availability

Data are available upon request to the guarantors of the work M.B. Bizino or H.J. Lamb.

## Abbreviations

^1^H-MRS: Proton magnetic resonance spectroscopy
ALT: Alanine aminotransferase
AST: Aspartate aminotransferase
GGT: γ-Glutamyl transferase
GLP-1: Glucagon-like peptide-1
GLP-1R: GLP-1 receptor
GLP-1RA: GLP-1 receptor agonist
HTGC: Hepatic triacylglycerol content
MAGNA VICTORIA: MAGNetic resonance Assessment of VICTOza efficacy in the Regression of cardiovascular dysfunction In type 2 diAbetes mellitus
MTGC: Myocardial triacylglycerol content
NASH: Non-alcoholic steatohepatitis
SAT: Subcutaneous adipose tissue
SUD: Sulfonylurea derivative
VAT: Visceral adipose tissue

## References

[1] van der Meer RW, Lamb HJ, Smit JW, de Roos A (2012). MR imaging evaluation of cardiovascular risk in metabolic syndrome. Radiology.

[2] Park HS, Lee K (2005). Greater beneficial effects of visceral fat reduction compared with subcutaneous fat reduction on parameters of the metabolic syndrome: a study of weight reduction programmes in subjects with visceral and subcutaneous obesity. Diabet Med.

[3] Levelt E, Mahmod M, Piechnik SK (2016). Relationship between left ventricular structural and metabolic remodeling in type 2 diabetes. Diabetes.

[4] Chaston TB, Dixon JB (2008). Factors associated with percent change in visceral versus subcutaneous abdominal fat during weight loss: findings from a systematic review. Int J Obes.

[5] Ben-Shlomo S, Zvibel I, Shnell M (2011). Glucagon-like peptide-1 reduces hepatic lipogenesis via activation of AMP-activated protein kinase. J Hepatol.

[6] Sharma S, Mells JE, Fu PP, Saxena NK, Anania FA (2011). GLP-1 analogs reduce hepatocyte steatosis and improve survival by enhancing the unfolded protein response and promoting macroautophagy. PLoS One.

[7] Parlevliet ET, Wang Y, Geerling JJ (2012). GLP-1 receptor activation inhibits VLDL production and reverses hepatic steatosis by decreasing hepatic lipogenesis in high-fat-fed APOE*3-Leiden mice. PLoS One.

[8] Noyan-Ashraf MH, Shikatani EA, Schuiki I (2013). A glucagon-like peptide-1 analog reverses the molecular pathology and cardiac dysfunction of a mouse model of obesity. Circulation.

[9] Bi Y, Zhang B, Xu W (2014). Effects of exenatide, insulin, and pioglitazone on liver fat content and body fat distributions in drug-naive subjects with type 2 diabetes. Acta Diabetol.

[10] Jendle J, Nauck MA, Matthews DR (2009). Weight loss with liraglutide, a once-daily human glucagon-like peptide-1 analogue for type 2 diabetes treatment as monotherapy or added to metformin, is primarily as a result of a reduction in fat tissue. Diabetes Obes Metab.

[11] Li CJ, Yu Q, Yu P (2014). Changes in liraglutide-induced body composition are related to modifications in plasma cardiac natriuretic peptides levels in obese type 2 diabetic patients. Cardiovasc Diabetol.

[12] Santilli F, Simeone PG, Guagnano MT (2017). Effects of liraglutide on weight loss, fat distribution, and beta-cell function in obese subjects with prediabetes or early type 2 diabetes. Diabetes Care.

[13] Shi Li, Zhu Jing, Yang Ping, Tang Xiaoqiang, Yu Wenlong, Pan Changjie, Shen Moyu, Zhu Dalong, Cheng Jinluo, Ye Xinhua (2017). Comparison of exenatide and acarbose on intra-abdominal fat content in patients with obesity and type-2 diabetes: A randomized controlled trial. Obesity Research & Clinical Practice.

[14] Suzuki D, Toyoda M, Kimura M (2013). Effects of liraglutide, a human glucagon-like peptide-1 analogue, on body weight, body fat area and body fat-related markers in patients with type 2 diabetes mellitus. Intern Med.

[15] Tang A, Rabasa-Lhoret R, Castel H (2015). Effects of insulin glargine and liraglutide therapy on liver fat as measured by magnetic resonance in patients with type 2 diabetes: a randomized trial. Diabetes Care.

[16] Jonker JT, de Mol P, de Vries ST (2013). Exercise and type 2 diabetes mellitus: changes in tissue-specific fat distribution and cardiac function. Radiology.

[17] Dutour A, Abdesselam I, Ancel P (2016). Exenatide decreases liver fat content and epicardial adipose tissue in patients with obesity and type 2 diabetes: a prospective randomized clinical trial using magnetic resonance imaging and spectroscopy. Diabetes Obes Metab.

[18] Iacobellis G, Mohseni M, Bianco SD, Banga PK (2017). Liraglutide causes large and rapid epicardial fat reduction. Obesity (Silver Spring).

[19] Bizino MB, Sala ML, de Heer P (2015). MR of multi-organ involvement in the metabolic syndrome. Magn Reson Imaging Clin N Am.

[20] Bizino MB, Jazet IM, Westenberg JJM (2019). Effect of liraglutide on cardiac function in patients with type 2 diabetes mellitus: randomized placebo-controlled trial. Cardiovasc Diabetol.

[21] de Heer P, Bizino MB, Lamb HJ, Webb AG (2016). Parameter optimization for reproducible cardiac (1) H-MR spectroscopy at 3 Tesla. J Magn Reson Imaging.

[22] Pastel E, McCulloch LJ, Ward R (2017). GLP-1 analogue-induced weight loss does not improve obesity-induced AT dysfunction. Clin Sci (Lond).

[23] Drucker DJ (2018). Mechanisms of action and therapeutic application of glucagon-like peptide-1. Cell Metab.

[24] Lee YS, Park MS, Choung JS (2012). Glucagon-like peptide-1 inhibits adipose tissue macrophage infiltration and inflammation in an obese mouse model of diabetes. Diabetologia.

[25] Kreier F, Kap YS, Mettenleiter TC (2006). Tracing from fat tissue, liver, and pancreas: a neuroanatomical framework for the role of the brain in type 2 diabetes. Endocrinology.

[26] Kreier F, Fliers E, Voshol PJ (2002). Selective parasympathetic innervation of subcutaneous and intra-abdominal fat--functional implications. J Clin Invest.

[27] Kooijman S, Wang Y, Parlevliet ET (2015). Central GLP-1 receptor signalling accelerates plasma clearance of triacylglycerol and glucose by activating brown adipose tissue in mice. Diabetologia.

[28] Yan Jinhua, Yao Bin, Kuang Hongyu, Yang Xubin, Huang Qin, Hong Tianpei, Li Yushu, Dou Jingtao, Yang Wenying, Qin Guijun, Yuan Huijuan, Xiao Xinhua, Luo Sihui, Shan Zhongyan, Deng Hongrong, Tan Ying, Xu Fen, Xu Wen, Zeng Longyi, Kang Zhuang, Weng Jianping (2019). Liraglutide, Sitagliptin, and Insulin Glargine Added to Metformin: The Effect on Body Weight and Intrahepatic Lipid in Patients With Type 2 Diabetes Mellitus and Nonalcoholic Fatty Liver Disease. Hepatology.

[29] Petit JM, Cercueil JP, Loffroy R (2017). Effect of liraglutide therapy on liver fat content in patients with inadequately controlled type 2 diabetes: the Lira-NAFLD Study. J Clin Endocrinol Metab.

[30] Armstrong MJ, Gaunt P, Aithal GP (2016). Liraglutide safety and efficacy in patients with non-alcoholic steatohepatitis (LEAN): a multicentre, double-blind, randomised, placebo-controlled phase 2 study. Lancet.

[31] Cuthbertson DJ, Irwin A, Gardner CJ (2012). Improved glycaemia correlates with liver fat reduction in obese, type 2 diabetes, patients given glucagon-like peptide-1 (GLP-1) receptor agonists. PLoS One.

[32] Ishii S, Iizuka K, Miller BC, Uyeda K (2004). Carbohydrate response element binding protein directly promotes lipogenic enzyme gene transcription. Proc Natl Acad Sci U S A.

[33] Rijzewijk LJ, van der Meer RW, Smit JW (2008). Myocardial steatosis is an independent predictor of diastolic dysfunction in type 2 diabetes mellitus. J Am Coll Cardiol.

[34] Hammer S, Snel M, Lamb HJ (2008). Prolonged caloric restriction in obese patients with type 2 diabetes mellitus decreases myocardial triglyceride content and improves myocardial function. J Am Coll Cardiol.

[35] Wu Lujin, Wang Ke, Wang Wei, Wen Zheng, Wang Peihua, Liu Lei, Wang Dao Wen (2018). Glucagon-like peptide-1 ameliorates cardiac lipotoxicity in diabetic cardiomyopathy via the PPARα pathway. Aging Cell.

[36] Leonardini A, D’Oria R, Incalza MA (2017). GLP-1 Receptor Activation Inhibits Palmitate-Induced Apoptosis via Ceramide in Human Cardiac Progenitor Cells. J Clin Endocrinol Metab.

[37] Levelt E, Pavlides M, Banerjee R (2016). Ectopic and visceral fat deposition in lean and obese patients with type 2 diabetes. J Am Coll Cardiol.

[38] Mahabadi AA, Berg MH, Lehmann N (2013). Association of epicardial fat with cardiovascular risk factors and incident myocardial infarction in the general population: the Heinz Nixdorf Recall Study. J Am Coll Cardiol.

[39] van Eyk HJ, van Schinkel LD, Kantae V (2018). Caloric restriction lowers endocannabinoid tonus and improves cardiac function in type 2 diabetes. Nutr Diabetes.

[40] Chau YY, Bandiera R, Serrels A (2014). Visceral and subcutaneous fat have different origins and evidence supports a mesothelial source. Nat Cell Biol.

[41] Marso SP, Daniels GH, Brown-Frandsen K (2016). Liraglutide and cardiovascular outcomes in type 2 diabetes. N Engl J Med.

[42] Goodman SN, Berlin JA (1994). The use of predicted confidence intervals when planning experiments and the misuse of power when interpreting results. Ann Intern Med.

[43] Davies MJ, Bergenstal R, Bode B (2015). Efficacy of liraglutide for weight loss among patients with type 2 diabetes: the SCALE diabetes randomized clinical trial. Jama.

