# Supplementary material for: Placebo-controlled randomised trial with liraglutide on magnetic resonance endpoints in individuals with type 2 diabetes: a pre-specified secondary study on ectopic fat accumulation
Source: Diabetologia. 2019 Nov 5;63(1):65–74. doi: 10.1007/s00125-019-05021-6 (PMC6890592; doi:10.1007/s00125-019-05021-6)
Supplement: Supplementary file 1 — (PDF 319 kb) [file 125_2019_5021_MOESM1_ESM.pdf]

Supplementary table 1: pre-defined endpoints of the MAGNA VICTORIA study, as registered in clinicaltrials.gov

Clinical trial registry NCT01761318 pre-defined endpoint	Endpoint synonym / abbreviation	Reported in [1]	Reported in current manuscript	Future manuscript
**Primary endpoints**				
Stroke volume	SV	x		
Ejection Fraction	EF	x		
Cardiac output	CO	x		
Cardiac index	CI	x		
Peak ejection rate	PER	x		
Early peak filling rate	Early transmitral peak flow rate (E)	x		
Early deceleration peak	peak deceleration of transmitral early peak flow (Edec)	x		
Atrial peak filling rate	Late transmitral peak flow rate (A)	x		
Early deceleration peak / Atrial peak filling rate (E/A ratio)		x		
Peak mitral annulus longitudinal motion	Early peak mitral annular septal tissue velocity (Ea)	x		
Left ventricular filling pressure (= early peak filling rate / peak mitral annulus longitudinal motion)	E/Ea	x		
**Secondary endpoints**				
Aorta and carotid vessel wall imaging; total vessel wall area				*
Aorta and carotid vessel wall imaging; average vessel wall thickness				*
Aorta and carotid vessel wall imaging; maximum vessel wall thickness				*
Aorta and carotid vessel wall imaging; vascular distensibility (pulse wave velocity)				x
Adipose tissue distribution: change from baseline of the ratio subcutaneous fat / visceral abdominal fat	VAT/SAT		x	
Total body fat				*

Epicardial fat volume			x	
Magnetic Resonance Spectroscopy of the heart	Myocardial triglyceride content / myocardial steatosis (MTGC)		x	
Magnetic Resonance Spectroscopy of the liver	Hepatic triglyceride content / liver fat / hepatic steatosis (HTGC)		x	
Magnetic Resonance Spectroscopy of the kidney	Renal triglyceride content / Renal adiposity / steatosis (RTGC)			x ($)
HBA1C		x	x	
Fasting blood glucose level				x
Myocardial T1 - mapping				x
**Other pre-specified endpoints**				
Anthropometric measurements: length, body weight and calculated BMI.		x	x	
Waist / hip ratio			x	
Systolic blood pressure		x		
Diastolic blood pressure		x		
Resting Energy Expenditure				x
Immunological analysis: Fluorescence-Activated Cell Sorting (FACS)				#
Immunological analysis: Peripheral Blood Mononuclear Cell isolation to analyze immunological activation and status of subjects. Both quantification of white blood cells (T-cells, B-cells, macrophages) and functional analysis				#
Fasting insulin level				x
Leptin				#
Glucagon				#
Adiponectin		x		
Cholesterylester transfer protein (CETP)				x
High Sensitive C Reactive Protein				#

Free Fatty Acids			x	
Cholesterol level (total, HDL and LDL)			x	
Liver function tests (ALT, AST, alkaline phosphatase, GGT)			x	
Triglycerides			x	
Quantitative insulin sensitivity index (QUICKI)				x
Albuminuria				x
Immunological analysis as assessed by RNA profiling				x
Metabolomics in urine and blood				#
Insulin dose		x	x	
Hypoglycaemic episodes				x

* aorta and vessel imaging, total body fat were not performed because these scans could not be included in the MRI protocol that was limited by a duration of 2 hours.
# due to budget restrictions these endpoints were not be examined.
$ we chose not to report renal triglyceride content in the current manuscript for several reasons. First, to date it is not known whether renal adiposity is a feature of type 2 diabetes mellitus / obesity. Second, the relationship between renal adiposity and diabetic nephropathy has not (yet) been established. Third, the measurement of renal triglyceride content is technically very challenging, and as yet has not been validated in humans. Therefore, we chose to report this endpoint in a separate manuscript.

Supplementary table 2: ectopic fat accumulation, with between group changes adjusted for stratifiers sex and insulin use

	**Liraglutide (n=23)**			**Placebo (n=26)**			**Mean (95% CI) changes from baseline (Liraglutide vs Placebo)**	***p***
	**Baseline**	**26 weeks**	**Mean (SD) change from baseline**	**Baseline**	**26 week**	**Mean (SD) change from baseline**		
Visceral fat, cm^2	207 (87)	203 (88)	-8 (33)	204 (63)	200 (55)	0 (27)	-7 (-24 to 10)	0.41
Subcutaneous fat, cm^2	361 (142)	339 (131)	-28 (40)	329 (107)	333 (125)	3 (30)	-31 (-53 to -9)	0.006
HTGC, %	18.1 (11.2)	12.0 (7.7)	-6.3 (7.1)	18.4 (9.4)	14.7 (10.0)	-4.0 (4.6)	-2.0 (-5.2 to 1.2)	0.22
MTGC, %	1.5 (0.6)	1.2 (0.6)	-0.3 (0.5)	1.3 (0.5)	1.2 (0.6)	-0.0 (0.5)	-0.1 (-0.4 to 0.2)	0.46
Epicardial fat, cm^2	8.9 (4.3)	9.1 (4.7)	0.3 (3.0)	9.6 (4.1)	9.6 (4.6)	0.0 (2.2)	0.1 (-1.5 to 1.8)	0.86
Paracardial fat, cm^2	25.7 (10.9)	24.7 (10.9)	-1.1 (6.0)	20.6 (10.0)	22.0 (10.3)	1.4 (5.7)	-1.8 (-5.3 to 1.7)	0.30
Pericardial fat, cm^2	34.6 (13.4)	33.8 (13.9)	-0.8 (7.4)	30.2 (12.3)	31.7 (12.4)	1.5 (5.7)	-1.8 (-6.0 to 2.4)	0.40

Supplementary table 3: stepwise multiple linear regression analysis for the dependent variable HTGC

Independent variable	Unadjusted estimate of association	95% confidence interval	p-value
Change in HbA1c, per 1 mmol/mol	0.28	0.12 to 0.44	0.001
Sex, men vs women	2.44	-0.91 to 5.79	0.15
Age, per 1 year	0.04	-0.23 to 0.30	0.78
Treatment allocation, liraglutide vs placebo	-0.72	-4.58 to 3.15	0.71
Weight loss, per 1 kg	0.43	-0.08 to 0.94	0.10

MAGNA VICTORIA study group:

The MAGNA VICTORIA study group assisted with selection of eligible patients. Participants of the study group are (in random order):

M.A. Diez Canseco Quintana, MD
Huisartsenpraktijk Smithuis en Diez Canseco
Gouda, The Netherlands

C. Overman, MD
Huisartsenpraktijk Overman
Leidschendam, The Netherlands

I. Minken, MD
Huisartsenpraktijk Minken
Leiden, The Netherlands

H. Laurier
Prelude Huisartsenpraktijk
Alphen aan den Rijn, The Netherlands

E. Pleij, MD
Prelude Huisartsenpraktijk
Alphen aan den Rijn, The Netherlands

M. de Winde
Huisartspraktijk Hoogh Teylingen
Voorhout, The Netherlands

T.N. Bonten, MD, PhD
Huisartspraktijk L. van Duijn
Katwijk, The Netherlands

L. van Duijn, MD
Huisartspraktijk L. van Duijn
Katwijk, The Netherlands

P.H.L.M. Geelhoed, MD, PhD
Haaglanden Medical Center
The Hague, The Netherlands

A.H. Bootsma, MD, PhD
Haaglanden Medical Center
The Hague, The Netherlands

A.V. Kharagjitsingh, MD, PhD

Haaglanden Medical Center
The Hague, The Netherlands

Reference list:

[1] Bizino MB, Jazet IM, Westenberg JJM, et al. (2019) Effect of liraglutide on cardiac function in patients with type 2 diabetes mellitus: randomized placebo-controlled trial. Cardiovascular diabetology 18(1): 55. 10.1186/s12933-019-0857-6
